# An efficient algorithm calculating common solvent accessible volume

**DOI:** 10.1371/journal.pone.0265614

**Published:** 2022-03-21

**Authors:** In Jung Kim, Hyuntae Na

**Affiliations:** Department of Computer Science, Penn State Harrisburg, Middletown, Pennsylvania, United States of America; Koç University, TURKEY

## Abstract

The solvent accessible surface area and the solvent accessible volume are measurements commonly used in implicit solvent models to include the effect of forces exerted by solvents on the protein surfaces (or the atoms on protein surfaces). The two measurements have limitations in describing interactions between proteins (or proteins’ atoms) mediated/bridged by solvents. This is because describing the interactions between proteins should be able to capture the chain of protein-solvent-protein interactions while the solvent accessible surface area or the solvent accessible volume can capture only protein-solvent interactions. If we represent the solvent as a continuous medium, we can consider an atom of a protein can effectively interact with the solvent within a certain distance from its surface (or its own solvent-interacting sphere). In this case, the protein-solvent-protein interactions can be measured by the amount of solvent interacting with two proteins’ atoms at the same time (or the volume shared by the two atoms’ solvent-interacting spheres excluding the volumes occupied by proteins’ atoms). We call the shared volume as the common solvent accessible volume (CSAV); there has been no method developed to determine the CSAV. In this work, we propose a new sweep-line-based method that efficiently calculates the common solvent accessible volume. The performance and accuracy of the proposed sweep-line-based method are compared with those of the naïve voxel-based method. The proposed method takes log-linear time to the number of atoms involved in a CSAV calculation and linear time to the resolution. Our results, tested with 52 protein structures of various sizes, show that the proposed sweep-line-based method is superior to the voxel-based method in both computational efficiency and accuracy.

## Introduction

Most proteins carry out their functions by interacting with other proteins or ligands, and these interactions are often mediated by solvents. Therefore, understanding the interactions between proteins/ligands mediated by solvents is key to accurately understanding the functional mechanisms of proteins and to designing drugs. Treating each solvent particle as an individual unit in simulations of protein-protein/ligand interactions provides a more realistic and accurate simulation condition, but it is computationally demanding. To reduce the computational cost and increase the speed of conformational samplings, often implicit solvent models have been used, such as the molecular mechanics generalized Born solvent accessible surface area (MM/GBSA) [1] or the molecular mechanics Poisson-Boltzmann solvent accessible surface area (MM/PBSA) [2]. Implicit solvent models treat solvents as a continuous medium rather than explicitly handling individual solvent particles, such as water molecules. In many cases, implicit solvent models are composed of the solvent accessible surface area (SASA) term and the continuum electrostatics term [3, 4]. The SASA term has been used to include the linear relationship between the solvation free energy and SASA [3] and to model the forces exerted on a protein’s atom by solvents [4]. The solvent accessible volume (SAV) has been used to substitute for or refine the SASA term, in order to include the influence of solvents on the protein’s interior [5–7]. The continuum electrostatics term models the protein-solvent electrostatic interactions, and it is usually described using the Poisson-Boltzmann (PB) or generalized Born (GB) equations.

However, there are some limitations in describing protein-protein interactions mediated by solvents using the implicit solvent models that use SASA and/or SAV. If we look at the interactions between proteins mediated by solvents at the atomic level, the interactions can be described as the cases that solvent particles bridge the gap between proteins’ atoms. The bridging solvent particles can (i) promote the interaction between hydrophilic residues or (ii) defer their direct interactions by forming protein-solvent-protein interactions or hydrogen-bond networks to provide extra time for proteins to rearrange their side chains [8, 9]. The bridging effect cannot be captured by SASA and SAV because they can measure only protein-solvent interactions; the bridging effect could be captured by a measurement describing protein-solvent-protein interactions. Note that a protein’s atom can have significantly meaningful interactions with solvent particles within 3.5 Å from the atom’s surface or sphere, which is called the hydration shell [10]. Consider the solvent as a continuous medium. Let the solvent-interacting sphere of an atom be the spherical range where the solvent can interact with the atom. The protein-solvent-protein interactions, or the solvent-mediated protein-protein interactions, can be estimated by the amount of solvent interacting with two proteins’ atoms at the same time (or the magnitude of non-bonded interaction of solvent with them). The amount of solvent, which is the simpler than the magnitude, is same to the volume shared by the two atoms’ solvent-interacting spheres excluding the volumes occupied by proteins’ atoms, or the common solvent accessible volume (CSAV) in short. The CSAV can be a useful term to improve the accuracy of implicit solvent models, especially in explaining the solvent-mediated protein-protein/ligand interactions.

Many methods have been developed to determine the protein surface area, the protein volume, and the solvent accessible volume [11–26]. Those methods are developed by using Voronoi diagrams [11–13], polyhedrons [14], the inclusion-exclusion principle [15–17] by finding the overlapping volume of three spheres [18, 19], the integration of the solvent accessible surface area [20, 21], or the voxels (or cubes) [22–26]. The methods determining the solvent accessible volume (SAV) have been used for the free energy calculation [12, 16, 17, 20, 27, 28]. However, these methods cannot be used to determine the common solvent accessible volume (CSAV). More specifically, the existing methods, especially those determining the protein volume or the solvent accessible volume, are designed to determine the volume of the union of spheres (representing atoms and solvent-interacting spheres). On the other hand, determining the CSAV requires both the intersection and exclusion of spheres: the CSAV is defined in terms of intersecting volume of two spheres (representing solvent-interacting spheres) after excluding other spheres (representing atoms) within the intersection volume. It is theoretically possible to determine the CSAV using the inclusion-exclusion principle and the existing methods determining the union of spheres. However, this approach is impractical because the inclusion-exclusion principle with *n* sets or spheres has 2^*n*^ − 1 number of terms. In other words, calculating the CSAV using the existing methods (determining the union of spheres) may require the union of all different combinations of spheres (2^n^ number of unions) in the worst-case scenarios.

In this work, we present a new method that efficiently determines the CSAV of two atoms. We determine the CSAV by numerically integrating the true cross-sectional area of the CSAV. The true cross-sectional area of the CSAV sliced by a plane is determined by dividing the area into several patches utilizing the sweep-line algorithm [29] and calculating the patches’ areas using a closed-form solution. Our results show that the proposed method determines the CSAV value in *O*(*mn* log *n*), where *n* is the number of atoms involved in the CSAV calculation and *m* is the resolution that controls the trade-off between the computational cost and the accuracy. We compare the proposed method with a naïve voxel-based method, which is related to other voxel-based methods [22–26] but is designed to determine the CSAV value. The results show that the proposed method is superior to the voxel-based method in both computational efficiency and accuracy.

## Methods

In this section, we define the common solvent accessible volume (CSAV) and describe a naïve voxel-based approach. Then, we introduce the proposed sweep-line-based method that improves the efficiency and accuracy of the CSAV calculation.

### Common Solvent Accessible Volume (CSAV)

**Fig 1:**
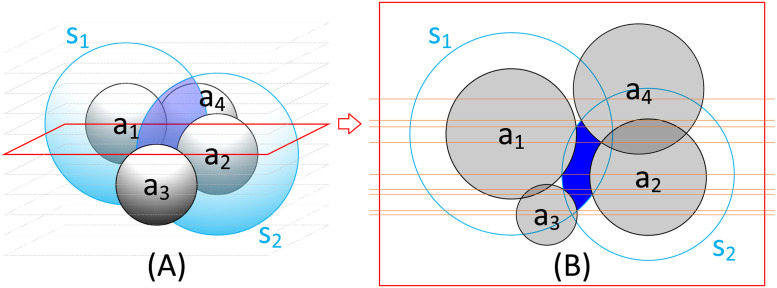
Illustration of the common solvent accessible volume of two atoms *a*_1_ and *a*_2_ in (A), and its cross-sectional shape and sweep lines in (B).

We define the solvent accessible volume (SAV) of a protein’s atom as the space where the solvent as a continuous medium can interact with the atom. More specifically, we define the SAV of an atom *a* as the volume of a sphere whose radius is *r*_*a*_ + *d*, excluding the space occupied by any atoms including *a*, where *r*_*a*_ is the radius of the atom *a* and *d* is the thickness of the solvent layer. If the value of *d* is 3.5 Å, then the SAV is the volume of the hydration shell of the atom [10]. The common solvent accessible volume (CSAV) is the shared volume of two atoms’ SAVs. The CSAV measures the space where the solvent can mediate the interactions between the two atoms. Fig 1 illustrates the CSAV of two atoms *a*_1_ and *a*_2_. In Fig 1(A), two light blue spheres illustrate the SAVs of *a*_1_ and *a*_2_, and the dark blue region highlights the CSAV of *a*_1_ and *a*_2_. Fig 1(B) shows the cross-sectional shape of the CSAV’s environment sliced by the red plane in (A), where the blue region highlights the cross-sectional area of the CSAV. The orange horizontal lines represent sweep lines, which will be explained later.

### The voxel-based method

Here, we introduce a naïve voxel-based (or cube-based) method that determines the CSAV value by counting voxels within the CSAV. Algorithm 1 determines the CSAV of two atoms *a*_1_ and *a*_2_. The algorithm takes as input the two atoms *a*_1_ and *a*_2_, the set of atoms {*a*_3_, …, *a*_*n*_} to exclude from the CSAV of *a*_1_ and *a*_2_, the thickness *d* of the solvent layer, and the voxel size *δ*. Lines 1–2 determine the coordinate **a**_*i*_ and the radius *r*_*i*_ of an atom *a*_*i*_ for all *n* atoms, where 1 ≤ *i* ≤ *n*. Denote by *s*_1_ and *s*_2_ the solvent-interacting spheres of atoms *a*_1_ and *a*_2_ whose centers are **a**_1_ and **a**_2_ and whose radii are *r*_1_ + *d* and *r*_2_ + *d*, respectively. Line 3 determines the boundary of the CSAV: the bounding box of the shared regions of *s*_1_ and *s*_2_. Lines 4–15 count the number *c* of voxels in the CSAV, whose size is *δ* × *δ* × *δ* Å^3^. Lines 8–9 determine if a voxel at (*x*, *y*, *z*) is in both *s*_1_ and *s*_2_. Lines 10–11 increase the number *c* when the voxel is not occupied by any atoms including *a*_1_ and *a*_2_. In the algorithm, ∥**v**∥ is the Euclidean norm of a vector **v**. In our experiment, before calling the algorithm, we select atoms in {*a*_3_, …, *a*_*n*_} that overlap with both *s*_1_ and *s*_2_, to eliminate the unnecessary computational cost and to ensure fair comparisons with the sweep-line-based method. This voxel-based method can provide an accurate CSAV as the voxel size *δ* gets smaller. Denote by *m* the resolution that is the number of voxels per Å or *m* = 1/*δ*. Note that the number of iterations to update *x*, *y*, and *z* in lines 5–7 is proportional to *m*^3^, and line 10 requires *n* iterations. Therefore, the computational cost of Algorithm 1 is *O*(*m*^3^ *n*). The efficiency and accuracy of this voxel-based method will be compared with those of the proposed sweep-line-based method in the Results section.

**Algorithm 1** Voxel-based Method(*a*_1_, *a*_2_, {*a*_3_, …, *a*_*n*_}, *d*, *δ*)

1: **a**_1_, **a**_2_, …, **a**_*n*_ ← coordinates of *a*_1_, *a*_2_, …, *a*_*n*_

2: *r*_1_, *r*_2_, …, *r*_*n*_← radii of *a*_1_, *a*_2_, …, *a*_*n*_

3: *x*_min_, *x*_max_, *y*_min_, *y*_max_, *z*_min_, *z*_max_← CSAV boundary of *a*_1_ and *a*_2_

4: *c* ← 0

5: **for** *x*← *x*_min_ **to** *x*_max_ **step** *δ* **do**

6:  **for** *y*← *y*_min_ **to** *y*_max_ **step** *δ* **do**

7:   **for** *z*← *z*_min_ **to** *z*_max_ **step** *δ* **do**

8:    **p** ← (*x*, *y*, *z*)

9:    **if** ∥**a**_1_ − **p**∥ ≤ *r*_1_ + *d* **and** ∥**a**_2_ − **p**∥ ≤ *r*_2_ + *d* **then**

10:     *b* ← ∀1 ≤ *i* ≤ *n*, ∃*i* such that ∥**a**_*i*_ − **p**∥ ≤ *r*_*i*_

11:     $c\leftarrow \{\begin{array}{cc} c+1 & \text{if}\;b\;\text{is}\;\text{false;}\\ c & \text{otherwise} \end{array}$

12:    **end if**

13:   **end for**

14:  **end for**

15: **end for**

16: *csav* ← *c* ⋅ *δ*^3^

17: **return** *csav*

### The sweep-line-based method

In this section, we propose the algorithm that efficiently and accurately determines the CSAV of two atoms by numerically integrating the true cross-sectional area of the CSAV. Note that Fig 1 illustrates the CSAV of two atoms *a*_1_ and *a*_2_. Fig 1(A) shows how the CSAV of *a*_1_ and *a*_2_ is calculated when 4 atoms *a*_1_–*a*_4_ are involved in this calculation. Fig 1(B) shows the cross-sectional shape of the CSAV sliced by the red plane in (A) from the top view: the shape is composed of two solvent circles (*s*_1_ and *s*_2_) and four atom circles (*a*_1_–*a*_4_). In the figure, the blue cross-sectional area of the CSAV is split into several smaller solvent patches by horizontal orange lines that are determined utilizing the sweep-line algorithm [29]. The blue cross-sectional area is determined by summing the solvent patches’ areas calculated using a closed-form solution that will be explained later. By numerically integrating the cross-sectional areas of the CSAV in Fig 1(A), the proposed method finally determines the CSAV value.

In the following sections, we describe details of the proposed algorithm in five steps. First, given information for the cross-sectional shape of the CSAV sliced by a plane, we determine the sweep lines that split the cross-sectional area into solvent patches, such as the orange lines in Fig 1(B). Second, we determine the solvent patches that belong to the CSAV, which are blue regions between two orange lines. Third, the areas of solvent patches are calculated using the closed-form solution. Fourth, we integrate all components to determine the CSAV value. Finally, we analyze the time complexity of the proposed sweep-line-based method.

#### Determining the cross-sectional area of the CSAV utilizing the sweep-line algorithm

**Fig 2:**
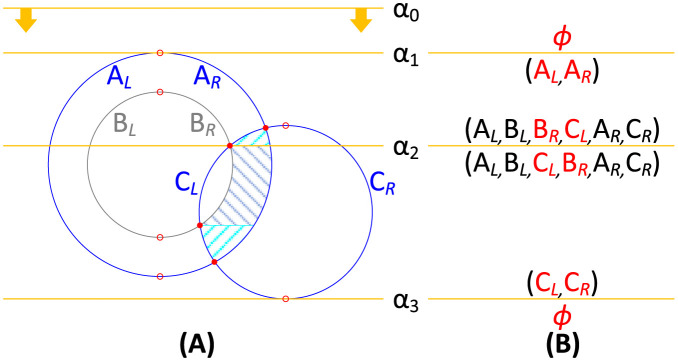
Illustration of how the sweep-line algorithm handles three different types of event points. (A) A cross-sectional plane that contains two solvent circles *A* and *C*, and one atom circle *B*. Small hollow (and filled) red circles represent circle-start and circle-end (and segment-intersection) event points. Three orange horizontal lines represent the sweep lines at the three different types of event points. Three light blue regions with hatch patterns show three solvent patches belonging to the cross-sectional area of the CSAV. (B) An illustration showing how the circle segments (*A*_*L*_, *A*_*R*_, …, *C*_*R*_) are stored in a sorted list before (above the orange sweep line) and after (below) handling events.

In this section, we describe how to determine the sweep lines that divide the cross-sectional area of the CSAV into solvent patches utilizing the sweep-line algorithm [29]. The sweep-line algorithm has been developed to efficiently identify all intersection points of *n* straight line segments in a plane in *O*(*n* log *n*) time. Note that atoms and solvent-interacting spheres (or solvent spheres in short) in a cross-sectional plane are represented as circles that are composed of left- and right-half-circle segments. We find intersection points between the curved circle segments utilizing the sweep-line algorithm. Fig 2 illustrates how the sweep-line algorithm works with the circle segments. While an (orange) horizontal sweep line slides down from the top (*α*_0_) to the bottom (*α*_3_), when the sweep line passes through (red) event points, the algorithm handles the events to maintain the sorted list of circle segments on the sweep line and to determine solvent patches for calculating the cross-sectional area of the CSAV. There are three event types: circle start, circle end, and segment-intersection. In Fig 2(A), *A* and *C* represent two solvent circles that are determined from solvent spheres, and *B* represents an atom circle that is determined from an atom. In the figure, *A*_*l*_ and *A*_*r*_ (and *B*_*l*_, *B*_*r*_, *C*_*l*_ and *C*_*r*_) represent the left- and right-circle segments of *A* (and *B* and *C*), respectively, and *α*_1_, *α*_2_ and *α*_3_ illustrate the three cases when the sweep line passes the circle-start, the segment-intersection and the circle-end event points, respectively. Fig 2(B) illustrates how the sorted list of circle segments on the sweep line is maintained before (above) and after (below) the orange sweep line as the sweep line passes through the three different types of event points. The red symbols in the list highlight that the list is modified locally by: (i) adding two circle segments into the list when their corresponding circle starts in *α*_1_, (ii) swapping two circle segments in the list when they intersect in *α*_2_, and (iii) removing two circle segments when their corresponding circle ends in *α*_3_.

Algorithm 2 describes the procedural steps of determining the cross-sectional area of the CSAV by iteratively creating/handling event points, maintaining the sorted list of circle segments, and sliding down the sweep line. The algorithm takes as input the information of two solvent circles $c_{s_{1}}$ and $c_{s_{2}}$ and a set of atom circles $\left\{c_{a_{1}},...,c_{a_{n}}\right\}$. In the algorithm, *e*_*i*_ is the *i*-th event point that the sweep line is currently passing through, *T* is the sorted list of circle segments that are on the sweep line, and *Q* is the event queue containing event points to be handled or below the sweep line. The two data structures *T* and *Q* are implemented as a balanced binary search tree and a priority queue, respectively. The algorithm first initiates the tree *T*, adds all circle-start and circle-end event points determined from all circles into the event queue *Q*, and sets the initial event point *e*_0_ as a point above all event points in *Q*. In line 7, the top-most point in *Q* is assigned to *e*_*i*_, which means that the sweep line slides down and passes the point *e*_*i*_ at the *i*-th iteration. Lines 8–9 determine solvent patches in between sweep lines determined by *e*_*i*−1_ and *e*_*i*_ and calculate their areas. Determining the solvent patches and calculating their areas will be described in the next two sections. Lines 10–29 handle the circle-start, circle-end, and segment-intersection event points by updating *T* and adding segment-intersection event points into *Q* if needed. The function AddSegmentIntersectionEvent(*Q*, *s*_1_, *s*_2_) inserts segment-intersection event points into *Q* when there are intersections between circles of the two segments *s*_1_ and *s*_2_, which will be explained in Algorithm 3. Lines 5–30 repeat the steps of popping an event point from *Q* and handling it to maintain the circle segments in *T*, until *Q* becomes empty. In line 17, if the circle of *e*_*i*_ is a solvent circle $c_{s_{1}}$ or $c_{s_{2}}$, the algorithm can return the *area* value to save computational cost by skipping unnecessary iterations; however, we continue the algorithm until *Q* is empty, in order to make a fair comparison with the voxel-based method in Algorithm 1. In degenerate cases where several event points are on the same sweep line, we select them in the following order: segment-intersection events, circle-end events, and circle-start events. Lines 24–28 handle the event point where only two circle segments intersect. However, when more than two circle segments intersect at the same point, which occurs only 0.00004% of the time in our experiment, we resolve this degenerate case in three steps as follows: (1) the circle segments are sorted by the slopes of their tangents at the point, (2) their locations in *T* are updated in the sorted order, and (3) the segment-intersection event points between their subsequent circle segments are added into *Q*.

**Algorithm 2** CrossSectionalAreaOfCSAV $\left(c_{s_{1}},c_{s_{2}},\left\{c_{a_{1}},...,c_{a_{n}}\right\}\right)$

1: *T*← an empty binary search tree

2: *Q*← a priority queue that contains all circle-start and

   circle-end event points of circles $\left\{c_{s_{1}},c_{s_{2}},c_{a_{1}},...,c_{a_{n}}\right\}$

3: *i* ← 0

4: *e*_*i*_ ← ∞

5: **while** *Q* is not empty **do**

6:  *i* ← *i* + 1

7:  *e*_*i*_ ← pop the top-most event point from *Q*

8:  *P* ← *SolventPatches*(*T*, *e*_*i*−1_, *e*_*i*_)

9:  *area* ← *area* + *PatchArea*(*P*, *e*_*i*−1_, *e*_*i*_)

10:  **if** *e*_*i*_ is a circle start event point **then**

11:   *s*_*l*_ and *s*_*r*_ ← left and right segments of circle of *e*_*i*_

12:   *T*.insert(*s*_*l*_)

13:   *T*.insert(*s*_*r*_)

14:   *s*_*p*_ and *s*_*s*_ ← *T*.predecessor(*s*_*l*_) and *T*.successor(*s*_*r*_)

15:   AddSegmentIntersectionEvent(*Q*, *s*_*p*_, *s*_*l*_)

16:   AddSegmentIntersectionEvent(*Q*, *s*_*r*_, *s*_*s*_)

17:  **else if** *e*_*i*_ is a circle end event **then**

18:   *s*_*l*_ and *s*_*r*_ ← left and right segments of circle of *e*_*i*_

19:   *s*_*p*_ and *s*_*s*_ ← *T*.predecessor(*s*_*l*_) and *T*.successor(*s*_*r*_)

20:   *T*.delete(*s*_*l*_)

21:   *T*.delete(*s*_*r*_)

22:   AddSegmentIntersectionEvent(*Q*, *s*_*p*_, *s*_*s*_)

23:  **else if** *e*_*i*_ is a segment intersection event **then**

24:   *s*_*l*_ and *s*_*r*_ ← left and right segments of *e*_*i*_

25:   *s*_*p*_ and *s*_*s*_ ← *T*.predecessor(*s*_*l*_) and *T*.successor(*s*_*r*_)

26:   *T*.swap(*s*_*l*_, *s*_*r*_) // interchange locations of *s*_*l*_ and *s*_*r*_ in *T*

27:   AddSegmentIntersectionEvent(*Q*, *s*_*p*_, *s*_*r*_)

28:   AddSegmentIntersectionEvent(*Q*, *s*_*l*_, *s*_*s*_)

29:  **end if**

30: **end while**

31: **return** *area*

Algorithm 3 describes the procedural steps of adding segment-intersection event points into the event queue *Q*. The algorithm takes as input the event queue *Q* and two circle segments *s*_*a*_ and *s*_*b*_. It assumes that the circle segment *s*_*a*_ is on the left side of the circle segment *s*_*b*_ in the sorted list: *s*_*a*_ is the predecessor of *s*_*b*_ in *T*. Lines 1–2 determine circles *c*_*a*_ and *c*_*b*_ of the circle segments *s*_*a*_ and *s*_*b*_, respectively. In lines 3–5 and 7, the algorithm skips adding segment-intersection events when (i) *c*_*a*_ and *c*_*b*_ are the same circle, (i) intersections between the two circles are already processed, (iii) the two circles intersect at a single point, or (iv) the intersection happens at the bottom of the circle *c*_*a*_ or *c*_*b*_. Lines 8–12 add a new segment-intersection event *e* into *Q* after making sure that *s*_1_ is on the left side of *s*_2_ above the point **p**.

**Algorithm 3** AddSegmentIntersectionEvent(*Q*, *s*_*a*_, *s*_*b*_)

1: *c*_*a*_ ← the circle of segment *s*_*a*_

2: *c*_*b*_ ← the circle of segment *s*_*b*_

3: **if** *c*_*a*_ ≠ *c*_*b*_or intersections between *c*_*a*_ and *c*_*b*_ are not yet processed **then**

4:  *P*← a set of intersection points between *c*_*a*_ and *c*_*b*_.

5:  **if** |*P*|≥2 **then**

6:   **for all** **p** ∈ *P* **do**

7:    **if** (**p** ≠ the circle-end point of *c*_*a*_) and (**p** ≠ the circle-end point of *c*_*b*_) **then**

8:     *s*_1_ ← the segment of *c*_*a*_ intersecting at **p**

9:     *s*_2_ ← the segment of *c*_*b*_ intersecting at **p**

10:     (*s*_1_, *s*_2_) ←(*s*_1_, *s*_2_) sorted by their tangent slope at **p**

11:     *e* ← a segment-intersection event of (*s*_1_, *s*_2_) at **p**

12:     *Q*.add(*e*)

13:    **end if**

14:   **end for**

15:  **end if**

16: **end if**

#### Determining solvent patches between sweep lines

**Fig 3:**
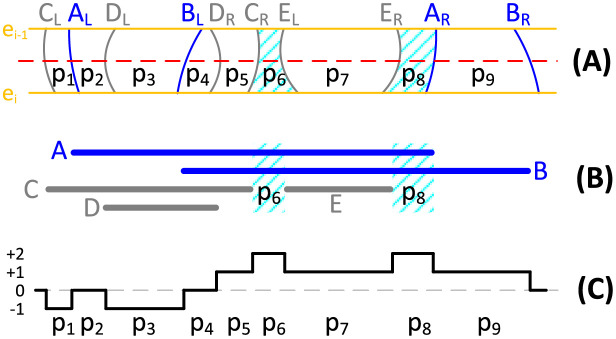
Illustration of procedure for finding solvent patches.

Let us define a patch as a small region that is surrounded by two adjacent circle segments and two consecutive sweep lines. The function SolventPatches(*T*, *e*_*i*−1_, *e*_*i*_) in line 8 of Algorithm 2 determines solvent patches that are patches on the CSAV and between two sweep lines determined by *e*_*i*−1_ and *e*_*i*_. We can determine if a patch is a solvent patch by counting the number of solvent circles and atom circles on it. Fig 3 illustrates the process of determining the solvent patches. Fig 3(A) shows the case where there are two blue solvent circles (*A* and *B*) and three gray atom circles (*D*, *E* and *F*) between two sweep lines determined by *e*_*i*−1_ and *e*_*i*_, and nine patches (*p*_1_, …, *p*_9_). Fig 3(B) shows the regions of solvent circles (two thick blue lines) and atom circles (three thick gray lines) on the red dashed line in Fig 3(A). Fig 3(C) depicts the values of the patches, each of which represents the number of solvent circles subtracted by the number of atom circles in the corresponding patch. In the figure, *p*_6_ and *p*_8_ are solvent patches: they belong to both solvent circles but no atom circles, and their values are 2.

Algorithm 4 determines the solvent patches between two consecutive sweep lines. The algorithm takes as input the two event points *e*_*i*−1_ and *e*_*i*_ representing the upper and lower sweep lines, respectively, and the tree *T* representing the sorted list of circle segments before handling *e*_*i*_ (or after handling *e*_*i*−1_). In the algorithm, *P* is the set of solvent patches, and *s*_*j*_ is the *j*-th circle segment in *T*, and *p*_*j*_ and *v*_*j*_ are the *j*-th patch and its value, respectively. Lines 3–12 scan circle segments in *T* from left to right. Line 6 determines the value *v*_*j*_ of the *j*-th patch by updating it from *v*_*j*−1_. Lines 7–11 add the *j*-th patch *p*_*j*_ into the set *P* when it is a solvent patch.

The function *SolventPatches*(*T*, *e*_*i*−1_, *e*_*i*_) in Algorithm 4 takes *O*(*k*) time, where *k* is the number of nodes in the tree *T*. However, this time complexity can be reduced as *O*(1). This is because the values of patches between sweep lines on *e*_*i*−1_ and *e*_*i*_ do not change from those on *e*_*i*−2_ and *e*_*i*−1_, except the values of patches that are adjacent to the two circle segments of *e*_*i*−1_. Therefore, *SolventPatches*(*T*, *e*_*i*−1_, *e*_*i*_) can take *O*(1) time by (i) storing the values of patches in the nodes of *T*, (ii) locally updating the values, and (iii) re-determining the solvent patches from those determined by *SolventPatches*(*T*, *e*_*i*−2_, *e*_*i*−1_). We skip the details of locally updating the values of patches and re-determining solvent patches in this paper: the local update is decided on a case-by-case basis depending on the type of event point *e*_*i*−1_ and the locations of *e*_*i*−1_’s circle segments in *T*.

**Algorithm 4** SolventPatches(*T*, *e*_*i*−1_, *e*_*i*_)

1: *P* ← ∅

2: *v*_0_ ← 0

3: *n* ← the number of segments in *T*

4: **for** *j* ← 1 to *n* − 1 **do**

5:  *s*_*j*_ ← the *j*-th element of *T*

6:  $v_{j}\leftarrow \{\begin{array}{cc} v_{j-1}+1 & \text{if}\;s_{j}\;\text{is}\;\text{a}\;\text{left}\;\text{solvent}\;\text{circle}\;\text{segment}\;\text{or}\;\text{a}\;\text{right}\;\text{atom}\;\text{circle}\;\text{segment}\\ v_{j-1}-1 & \text{if}\;s_{j}\;\text{is}\;\text{a}\;\text{right}\;\text{solvent}\;\text{circle}\;\text{segment}\;\text{or}\;\text{a}\;\text{left}\;\text{atom}\;\text{circle}\;\text{segment} \end{array}$

7:  **if** *v*_*j*_ = 2 **then**

8:   *s*_*j*+1_ ← the (*j* + 1)-th element of *T*

9:   *p*_*j*_ ← a solvent patch determined by (*s*_*j*_, *s*_*j*+1_, *e*_*i*−1_, *e*_*i*_)

10:   *P* ← *P* ∪ *p*_*j*_

11:  **end if**

12: **end for**

13: **return** *P*

#### Calculating areas of solvent patches

The function PatchArea(*P*, *e*_*i*−1_, *e*_*i*_) in line 9 of Algorithm 2 determines the total area of solvent patches determined by Algorithm 4. Note that each solvent patch is surrounded by two circle segments and two upper and lower sweep lines. Here, we derive the closed-form solution that calculates the area of a solvent patch.

Let (0, 0) and *r* be the center of a circle and its radius, respectively. The area *f*(*r*, *y*_0_, *y*_1_) of its right semicircle surrounded by the *y*-axis and two horizontal lines *y* = *y*_0_ and *y* = *y*_1_ can be calculated as follows:

$$\begin{array}{c} f\left(r,y_{0},y_{1}\right)=\int_{y_{0}}^{y_{1}}\sqrt{r^{2}-y^{2}}\;dy=g\left(r,y1\right)-g\left(r,y0\right)\;, \end{array}$$ (1)

where −*r* ≤ *y*_0_ < *y*_1_ ≤ *r* and

$$\begin{array}{c} g\left(r,y\right)=\int \sqrt{r^{2}-y^{2}}\;dy=\frac{1}{2}(y\sqrt{r^{2}-y^{2}}+r^{2}\text{arctan}(\frac{y}{\sqrt{r^{2}-y^{2}}}))\;. \end{array}$$ (2)

In the above equation, we select *π*/2 and −*π*/2 for $\text{arctan}(y/\sqrt{r^{2}-y^{2}})$ when *y* ≥ *r* and *y* ≤ −*r*, respectively. Using Eq (1), the area of a solvent patch surrounded by two circle segments and two upper- and lower-sweep lines can be calculated algebraically.

**Fig 4:**
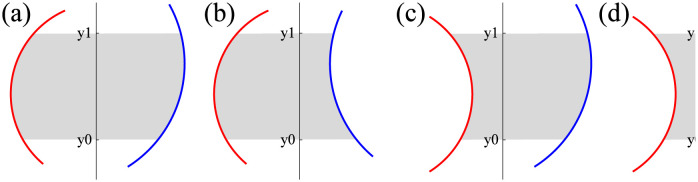
Four scenarios in which a gray solvent patch is surrounded by two circle segments and two sweep lines.

Fig 4 shows four different scenarios where a solvent patch (gray) is surrounded by two circle segments on its left- (red) and right-sides (blue). Let *l* and *r* be the circle segments on the left- and right-sides of a solvent patch, respectively, and *y*_0_ and *y*_1_ be the lower and upper bounds of the solvent patch (or two consecutive sweep lines *y* = *y*_0_ and *y* = *y*_1_), respectively. Denote by (*l*_*x*_, *l*_*y*_) and *l*_*r*_ the center of the circle segment *l* and its radius, respectively. In a similar manner, denote by (*r*_*x*_, *r*_*y*_) and *r*_*r*_ those of the circle segment *r*. The area *A*(*l*, *r*, *y*_0_, *y*_1_) of each gray patch in Fig 4 is calculated using the following formula:

$$\begin{array}{c} \begin{array}{cc} A\left(l,r,y_{0},y_{1}\right)=\left(y_{1}-y_{0}\right)\left(r_{x}-l_{x}\right) & -h\left(l\right)\cdot f\left(l_{r},y_{0}-l_{y},y_{1}-l_{y}\right)\\ & +h\left(r\right)\cdot f\left(r_{r},y_{0}-r_{y},y_{1}-r_{y}\right)\;, \end{array} \end{array}$$ (3)

where *h*(*s*) is a sign function that returns −1 and +1 if *s* is the left- and right-circle segments of a circle centering at (*s*_*x*_, *s*_*y*_), respectively.

Algorithm 5 calculates the total area of solvent patches. The algorithm takes as input the set *P* of solvent patches and two event points *e*_*i*−1_ and *e*_*i*_. Lines 3–4 determine the upper *y*_0_ and lower *y*_1_ bounds of solvent patches from *e*_*i*−1_ and *e*_*i*_, respectively, and the circle segments on left-side *l* and right-side *r* of the solvent patch *p*. Line 5 determines the area of a solvent patch *p* ∈ *P* using Eq (3) and adds its value to the total area.

**Algorithm 5** PatchArea(*P*, *e*_*i*−1_, *e*_*i*_)

1: *area* ← 0

2: **for all** *p* ∈ *P* **do**

3:  *l* and *r* ← circle segments on left- and right-side of *p*

4:  *y*_0_ and *y*_1_ ← *y*-axes of *e*_*i*−1_ and *e*_*i*_

5:  *area* ← *area* + *A*(*l*, *r*, *y*_0_, *y*_1_)

6: **end for**

7: **return** *area*

#### Determining the common solvent accessible volume (CSAV)

We integrate all the above algorithms to determine the common solvent accessible volume (CSAV) of two atoms. As illustrated in Fig 1, the CSAV of two atoms is determined by using the cross-sectional area of the CSAV sliced by a plane. To this end, we gradually slide down a cross-sectional plane that is parallel to the *xy*-plane. While sliding down the plane, we determine (i) atom circles and solvent circles to build the cross-sectional shape, (ii) the cross-sectional area of the CSAV from the circles, and (iii) the CSAV by numerically integrating the areas. Algorithm 6 determines the CSAV of two atoms using the numerical integration efficiently. The algorithm takes as input the two atoms *a*_1_ and *a*_2_, the set of atoms {*a*_3_, …, *a*_*n*_} to exclude from the CSAV of *a*_1_ and *a*_2_, the thickness *d* of the solvent layer, and the gap *δ* between cross-sectional planes. The algorithm first determines the solvent spheres *s*_1_ and *s*_2_ from the atoms *a*_1_ and *a*_2_ by increasing their radii by *d*, respectively, and the maximum (top) and minimum (bottom) *z*-coordinates of their shared solvent region of *s*_1_ and *s*_2_ in lines 1–2. Lines 6–19 slide down the cross-sectional plane *z* = *w* from top to bottom. Lines 8–11 determine the set *A* of atoms that intersect with the cross-sectional plane. Lines 12–14 determine two solvent circles $c_{s_{1}}$ and $c_{s_{2}}$ and a set *C* of atom circles. The two solvent circles are formed by the intersections of solvent spheres and the cross-sectional plane, and each atom circle in *C* is formed by the intersections of an atom in *A* and the cross-sectional plane. To avoid unnecessary computations in Algorithm 2, line 15 neglects atom circles in *C* if they do not overlap with both $c_{s_{1}}$ and $c_{s_{2}}$ and if they are fully enclosed by other atom circles. Lines 16–18 determine the cross-sectional area of the CSAV using Algorithm 2, accumulate its corresponding volume determined using the numerical integration into the total volume, and slide down the cross-sectional plane by *δ*. The source code that determine the CSAV using sweep-line algorithm is made publicly available in S1 File and at https://github.com/htna/CSAV.

**Algorithm 6** CalculateCSAV(*a*_1_, *a*_2_, {*a*_3_, …, *a*_*n*_}, *d*, *δ*)

1: *s*_1_ and *s*_2_ ← cloned spheres of *a*_1_ and *a*_2_ with increasing their radii by *d*

2: *w*_max_ and *w*_min_ ← max and min *z*-coordinates of shared region of *s*_1_ and *s*_2_

3: *W* ← a list of max and min *z*-coordinates of atoms {*a*_1_, …, *a*_*n*_}

4: *A* ← ∅

5: *csav* ← 0

6: **while** *w* ← *w*_max_

7: **while** *w* ≥ *w*_min_ **do**

8:  **while** *w* ≤ max(*W*) **do**

9:   *top* ← pop max(*W*) value from *W*

10:   $A\leftarrow \{\begin{array}{cc} A\cup \{a\} & \text{if}\;top\;\text{is}\;\text{the}\;\text{max}\;z\text{-coordinate}\;\text{of}\;\text{an}\;\text{atom}\;a\\ A\backslash \{a\} & \text{if}\;top\;\text{is}\;\text{the}\;\text{min}\;z\text{-coordinate}\;\text{of}\;\text{an}\;\text{atom}\;a \end{array}$

11:  **end while**

12:  $c_{s_{1}}$ ← a circle intersecting a sphere *s*_1_ and a plane *z* = *w*

13:  $c_{s_{2}}$ ← a circle intersecting a sphere *s*_2_ and a plane *z* = *w*

14:  *C* ← {*c*_*a*_∣ *c*_*a*_ is a circle intersecting a sphere *a* ∈ *A* and a plane *z* = *w*}

15:  *C* ← {*c*_*a*_∣*c*_*a*_ ∈ *C* overlaps with both $c_{s_{1}}$ and $c_{s_{2}}$, ∄*c*′ ∈ *C* s.t. *c*′ fully encloses *c*_*a*_}

16:  *area* ← CrossSectionalAreaOfCSAV $\left(c_{s_{1}},c_{s_{2}},C\right)$

17:  *csav* ← *csav* + *area* ⋅ *δ*

18:  *w* ← *w* − *δ*

19: **end while**

20: **return** *csav*

#### Running time analysis

Here, we provide the time complexity of Algorithm 6. Note that each atom can overlap with a limited number of other atoms because of the van der Waals force that prevents the collapse of any two atoms unless they are connected by bonds. This indicates that each atom circle representing the atom in the cross-sectional plane intersects with a constant (or limited) number of other atom circles on average. Assume that there are *n* atoms involved in the CSAV calculation, and the gap between cross-sectional planes is *δ* Å. For simplicity in the running time analysis, without loss of generality, assume that all atoms have the same radii and are uniformly distributed into a cube whose top face is parallel to the cross-sectional planes. Since each cross-sectional plane intersects *O*(*n*^2/3^) atoms, there are *O*(*n*^2/3^) circles in the plane and *O*(*n*^1/3^) circles in a sweep line. Recall that the tree *T* in Algorithm 2 represents the list of circle segments on the sweep line, and the tree *T* is updated by handling circle-start, circle-end, and segment-intersection events. In the algorithm, updating the tree *T* by each event takes *O*(log *n*^1/3^) time. Determining the solvent patches between two subsequent sweep lines in Algorithm 4 and determining their areas in Algorithm 5 take a constant time. This is because we update the values of patches and select the solvent patches locally only at the event point in Algorithms 4 and 5, respectively, and also because there are only 0–2 solvent patches between sweep lines in most cases (or 0.77 solvent patches on average) in Algorithm 5, which will be discussed in the Results section again. Since a circle intersects with a constant number of other circles on average in the cross-sectional plane, there are *O*(*n*^2/3^) event points, and the algorithm takes *O*(*n*^2/3^(log *n*^1/3^ + 1)) = *O*(*n*^2/3^log *n*) time to determine the cross-sectional area of the CSAV. Finally, determining the CSAV using Algorithm 6 takes *O*(*n*^2/3^ log *n* ⋅ *n*^1/3^/*δ*) = *O*(*mn* log *n*) time, where *m* is the resolution whose value is 1/*δ*. The resolution *m* controls the trade-off between the computational cost and the accuracy. Our results in the Results section show that the average running time of the proposed sweep-line-based method agrees with our time complexity analysis.

If we assume that all atoms have the same radii and are uniformly distributed in 3D space, the number *n* of atoms involved in the CSAV calculation should be proportional to the cube of the thickness *d* of the solvent layer. However, our results show that *n* is about proportional to the square of *d*: *n* ∝ *d*^1.9^. This is probably because the shared region of the two solvent spheres has a torus- or disk-like shape after excluding the two atoms’ regions and because the CSAV calculation only involves atoms overlapping with the torus- or disk-like shape. As a result, the average running time of the proposed sweep-line-based method can be written, using the thickness *d* of solvent layer, as *O*(*md*^2^ log *d*).

Note that the running time of the proposed sweep-line-based method increases linearly with the resolution *m*. In contrast to the sweep-line-based method, the running time of the naïve voxel-based method in Algorithm 1 increases cubically with the resolution *m*, that is *O*(*m*^3^ *n*) or *O*(*m*^3^ *d*^2^) where the voxel size is *δ* = 1/*m*. We will further discuss the running times of the sweep-line-based and the voxel-based methods in the Results section.

## Results

In this section, we evaluate the accuracy and efficiency of the proposed sweep-line-based method described in Algorithms 2–6 by comparing them to those of the naïve voxel-based method in Algorithm 1. In our work, the whole computation is performed using Intel(R) Xeon(R) CPU, whose clock speed is 2.20 GHz.

### Dataset preparation

We used 52 structures of proteins with 22–481 residues to test the two methods. To decide the 52 protein structures, we initially selected the 100 protein structures listed in the dataset used by Georgiev *et al*. [26]. The dataset includes proteins whose sequences are not redundant with a BLAST p-value cutoff of 10^−7^, which are selected arbitrarily using the VAST server. We additionally included two protein structures into the initial selection: Ubiquitin (pdb-id: 1UBQ) and Trp-cage (pdb-id: 3UC7, one of the smallest proteins). Given a protein structure among the 102 structures, we first updated the protein structure by adding missing atoms, such as hydrogens, and by removing water and ligand molecules using the Tinker program [30]. If the Tinker program fails to add missing atoms into the structure, we discarded it from our dataset. Finally, the 52 protein structures are selected and used to evaluate the accuracy and efficiency of the sweep-line-based and voxel-based methods. Table 1 shows the final list of the 52 protein structures used in this study. In the table, the first column shows pdb-ids and chain identifiers of the protein structures, and the second and third columns show the sizes of proteins in terms of the number of residues and atoms, including hydrogens, respectively.

**Table 1:** List of proteins used in this study.

pdb-id	resi^a^	atom^b^	pdb-id	resi	atom	pdb-id	resi	atom
3UC7A	22	283	1AG4A	103	1577	3KLOA	217	3561
1A11A	25	390	2NLLB	103	1709	4FAYA	227	3208
1S9KE	52	901	3DJ9A	107	1677	2OQ1A	251	3980
1ZLMA	58	930	2ETZA	108	1760	1ZHNA	270	4254
2ADRA	60	998	2CSHA	110	1696	4FYYA	310	4848
1DEMA	60	1003	2QHLC	111	1782	2A3VB	320	5353
1G2BA	62	966	2APFA	112	1688	1M2RA	327	5455
2TRCG	68	1132	2C4FU	116	1856	1A2OA	347	5299
2J7ZA	68	1148	1DX5I	118	1634	1EFYA	350	5591
1UBQA	76	1232	1NA0A	119	1853	4DNUA	366	5385
2ED0A	78	1152	3UBUB	125	2021	1MUWA	386	5168
1CL7I	82	1215	1YPQB	135	2106	1V6SA	390	5971
2YUQA	85	1349	1IT2A	146	2415	1AF6A	421	6426
2K0XA	86	1277	2DBAA	148	2189	1HQSA	422	6280
2JXBA	86	1446	1914A	171	2825	3KQNA	437	6536
1GVPA	87	1385	2GF5A	191	3036	1R64A	481	7255
1P47A	87	1461	2BNUA	203	3063			
1R1PB	100	1647	1PN9A	209	3274			

^a^The number of residues;

^b^The number of atoms including hydrogens.

We performed the following steps to decide the list of atom pairs to determine their common solvent accessible volumes (CSAVs) and to compare the accuracy and efficiency of the proposed sweep-line-based and voxel-based methods. First, note that two atoms do not have CSAV when they are apart or when they are located inside of proteins, assuming there is no cavity in the protein structures. Given a structure updated by adding missing atoms including hydrogens, we selected pairs of atoms satisfying the following conditions: (i) the distance between two atoms’ centers is smaller than 5 Å, and (ii) the distance between the surface of each atom and the surface of the atom’s protein is smaller than 3 Å. The van der Waals radius of each atom in the Chemistry at Harvard Macromolecular Mechanics (CHARMM) force field [31] is used as the radius of the atom. The protein surface is determined by rolling a ball whose probe radius is 1.4 Å using the method developed by Rashin et al [22]. We finally selected around 2.3 millions of atom pairs to determine their CSAVs from the 52 protein structures. The sweep-line-based and voxel-based methods can determine the CSAVs between atoms in different proteins, but we used the two methods to determine the CSAVs between atoms in one protein. This is because we are focusing on evaluating the accuracy and efficiency of the two methods in this experiments.

To evaluate the accuracy and efficiency of the two methods in different conditions of CSAVs, we used 16 different solvent layer thicknesses ranging 0.5–8.0 Å, which are the distances between the surface of an atom and solvent particles. Additionally, we used 19 different values for *δ* ranging 0.01–1 Å, which represent the gaps between cross-sectional planes or the voxel sizes. The gap or the voxel size *δ* is same to the inverse of resolution *m*, which will be discussed later: *δ* = 1/*m*.

### Statistics of atoms, circles, circle segments, and solvent patches in a CSAV calculation

Determining CSAVs is a complicated task since they have complex geometries. We collected statistics that show this complexity, including the number of atoms involved in CSAV calculations, the number of circles in a cross-sectional plane, and the number of circle segments in a sweep line. The statistics are collected from the 52 protein structures with a 3.5 Å thickness of the solvent layer and a 0.1 Å gap between cross-sectional planes. Note that the statistics are dependent on the thickness of the solvent layer, while the shape of their overall distribution will be similar to those of our results.

**Fig 5:**
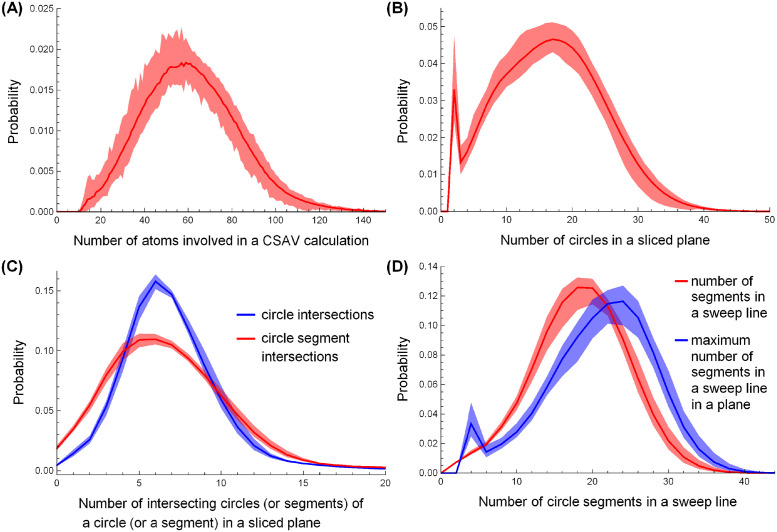
The statistics of the number of atoms, circles, circle segments, and intersections. The probability distribution averaged over the 52 proteins of (A) the number of atoms in a CSAV calculation, (B) the number of circles in a cross-sectional plane, (C) the number of intersections between circles (and circle segments) in a cross-sectional plane in red (and blue), and (D) the number (and the maximum number) of circle segments in a sweep line in red (and blue). The pink and light blue bands represent the range between the 5th and 95th percentiles of the red and blue probabilities determined from the 52 proteins, respectively.

Fig 5 shows the probability distributions of the number of atoms, circles, intersections, and circle segments involved in a CSAV calculation. In the figure, each (red or blue) solid line represents the probability averaged over 52 proteins’ probabilities, and each (pink or light blue) band represents the range between the 5th and 95th percentiles of the 52 probabilities. Fig 5(A) shows the probability distribution of the number of atoms involved in a CSAV calculation. The probability is calculated as follows. For a given protein, the number of atoms involved in each CSAV calculation is collected, and its normalized histogram is determined. After collecting all 52 normalized histograms of the proteins, their average and the range between the 5th and 95th percentiles are plotted as a red line and a pink band in the figure, respectively. The figure shows that a CSAV calculation involves around 61 atoms on average and around 140 atoms in the worst case. Fig 5(B)–5(D) are determined in a similar manner to Fig 5(A).

Fig 5(B) shows the probability distribution of the number of circles in a cross-sectional plane. The figure shows that a cross-sectional plane has around 17 circles on average and about 40 in the worst case. The sharp peak of the probability at 2 circles in the figure indicates that many cross-sectional planes contain only 2 solvent circles. The shared area between 2 solvent circles can be easily calculated algebraically; however, we did not use the algebraic solution in our experiments to make a fair comparison between the sweep-line-based method and the voxel-based method regarding both computational cost and accuracy. The figure shows higher probabilities with 3–16 circles than those with 18–31 circles, which are mainly caused by neglecting unnecessary circles in line 15 in Algorithm 6.

Fig 5(C) shows the probability distribution of the intersections between circles (red) and those between circle segments (blue) in a cross-sectional plane. It shows that the pink and light blue bands are very narrow, and there is an average of 6.9 intersections between circles and an average of 6.9 intersections between circle segments in a cross-sectional plane. This indicates that a circle (and a circle segment) overlaps with around 7 other circles (and circle segments) on average, independent of the protein size. From Fig 5(B) and 5(C), we can estimate the average number of event points in a cross-sectional plane. Our detailed inspection shows that there are 149 event points in a cross-sectional plane on average and around 270 event points in the 99th percentile, which agrees with our estimation.

Fig 5(D) shows the probability distribution of the number of circle segments on a sweep line. In the figure, the red line represents the probability of the number of circle segments on a sweep line while it slides down from top to bottom in a cross-sectional plane, and the pink band represents the range between the 5th and 95th percentiles. In a similar manner, the blue line and the light blue band represent the probability of the maximum number of circle segments that a sweep line can have in a cross-sectional plane. The figure shows that the sweep line interacts with an average of 18.4 circle segments and a maximum of 56 circle segments in all CSAVs computations, which corresponds to the average and maximum number of nodes in the tree *T* in Algorithm 2.

Table 2 shows that, in most cases, there are only 0–2 solvent patches on the sweep line in most cases even though the average tree size is 18 and the maximum tree size is 56. In our implementation of Algorithm 4, we keep the values of patches in their corresponding tree nodes, update only values of patches involved with the event point that is being handled, and keep the solvent patches in a separate list to avoid re-determining them when updating the values of patches. This enables determining the solvent patches in a sweep line and calculating their areas in *O*(1) time in Algorithm 4 and Algorithm 5, respectively, rather than taking *O*(*k*) time, where *k* is the number of circle segments in a sweep line.

**Table 2:** The probability of the number of solvent patches in a sweep line.

The number of solvent patches in a sweep line	0	1	2	3
Probability	0.41	0.43	0.14	0.02

**Fig 6:**
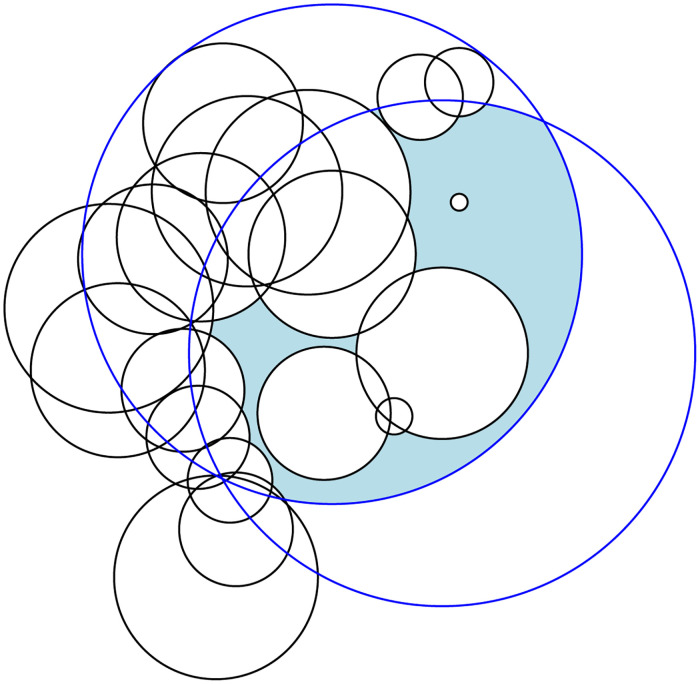
A snapshot of a cross-sectional shape that includes 21 circles.

Fig 5 shows the case in which the CSAV calculation involves an average of 60 atoms and its cross-sectional plane contains 17 circles, 18 nodes in a binary search tree, and 149 event points on average. Table 2 shows that there are usually 0–2 solvent patches on a sweep line. Fig 6 shows the snapshot of a cross-sectional shape that includes 21 circles. In the figure, 2 blue circles are solvent circles, 19 black circles are atom circles, and the light blue region is the cross-sectional area of the common solvent accessible volume of two atoms.

### Factors of CSAV computations

The accuracy and computational cost of calculating CSAV depend on several factors. The factors include the number of atoms involved in a CSAV calculation, the thickness of the solvent layer, and the gap between cross-sectional planes (or the resolution).

#### The number of atoms involved in a CSAV calculation

**Fig 7:**
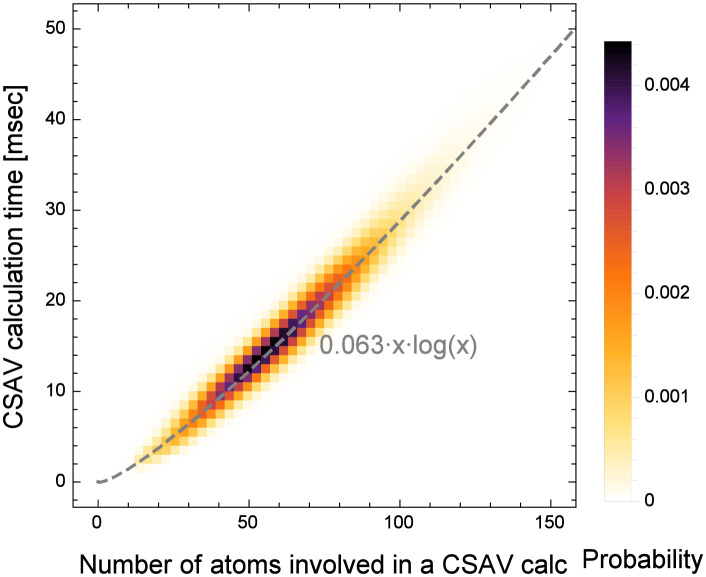
The probability of calculating a CSAV in *y* time when *x* number of atoms are involved in the calculation. The dashed line is the least-square fit of the log-linear function to the probability.

Fig 7 shows the relationship between the CSAV computation time and the number of atoms involved in the calculation. The figure is determined from all CSAV data collected from the 52 proteins with a 3.5 Å thickness of the solvent layer and a 0.1 Å gap between cross-sectional planes (or the resolution of 10 Å^–1^). In the figure, the value of each pixel represents the probability of determining CSAVs whose computations involve *x* number of atoms and require *y* milliseconds. The gray dashed line shows the least-square fit of the log-linear function to the probability distribution: *y* = 0.063*x* log *x*. Note that the coefficient, such as 0.063, is subject to change depending on the computing environments, such as the CPU type and the number of tasks running on the computer. Even though it seems that the CSAV computation time increases almost linearly with the number of atoms involved in the figure, our detailed inspection shows that there is a log-linear relationship. This also agrees with our time complexity analysis in the Methods section.

#### The thickness of the solvent layer and the CSAV computation time

**Fig 8:**
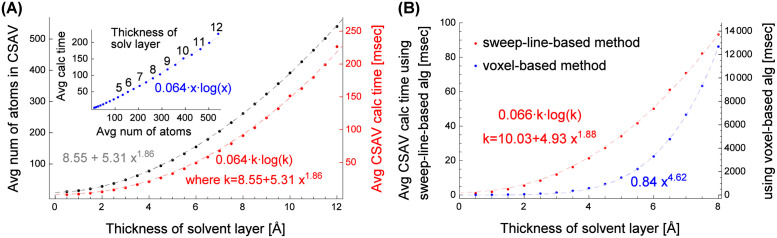
The relationship between the thickness of the solvent layer and the computational cost of sweep-line-based and voxel-based methods. (A) The relationship between the thickness of the solvent layer, the number of atoms involved in the CSAV calculation, and the CSAV computation time of the sweep-line-based method. (B) The comparison of the computation time of the sweep-line-based method and that of the voxel-based method when the thickness of the solvent layer increases.

Fig 8 shows the relationship between the thickness of the solvent layer, the number of atoms involved in the CSAV calculation, and the computational costs of the sweep-line-based method and the voxel-based method. Fig 8(A) shows the relationships between the thickness, the number of atoms, and the computation time of the proposed sweep-line-based method in milliseconds. The figure is determined from 1UBQ with a 0.1 Å gap between cross-sectional planes and the 16 different solvent layer thicknesses ranging 0.5–8.0 Å: 0.5, 1.0, 1.5, …, 8.0 Å. In the figure, each black point represents the average number of atoms in CSAV calculations in the *y*-axis when the solvent layer is *x* Å thick. In a similar manner, each red point represents their average computation time in milliseconds. The inset shows the same results in a different view: each blue point represents the solvent layer whose value is given above the point, where its *x* and *y* values represent the average CSAV computation time in milliseconds and the average number of atoms involved in the calculations, respectively. The gray dashed curve represents the least-square fit of $a_{1}+a_{2}x^{a_{3}}$ to black points. In a similar manner, the pink dashed curve represents that of *bk* log *k* where $k=a_{1}+a_{2}x^{a_{3}}$ to red points, and the light blue dashed curve represents that of *cx* log *x* to blue points.

Fig 8(B) compares the CSAV computation time of the sweep-line-based method and that of the voxel-based method when the thickness of the solvent layer increases. The figure is determined from 1ZLM with the 16 different solvent layer thicknesses ranging 0.5–8.0 Å and a 0.1 Å gap between cross-sectional planes (red) and a voxel size of 0.1 Å (blue). In the figure, red points represent the average CSAV computation time determined using the sweep-line-based method in milliseconds in the *y*-axis when the solvent layer is *x* Å thick. In a similar manner, blue points are determined using the voxel-based method when the voxel size is *x* × *x* × *x* Å^3^. The light blue dashed curve represents the least-square fit of $a_{1}x^{a_{2}}$ to blue points. The pink dashed curve represents the least-square fit of *bk* log *k* to red points, where *k* approximates the average number of atoms when the solvent layer is *x* Å thick; this curve is determined in the same way the pink dashed curve in Fig 8(A) is determined.

Fig 8 shows many interesting points. First, regarding the proposed sweep-line-based method, the number of atoms involved in the CSAV calculation increases near quadratically to the thickness of the solvent layer. The least-square fits of *k* = 8.55 + 5.31*x*^1.86^ in Fig 8(A) and *k* = 10.03 + 4.93*x*^1.88^ in Fig 8(B) are independently determined from 1UBQ and 1ZLM, respectively, which approximates the number of atoms *k* from the thickness of the solvent layer *x*. The two least square fits approximate to the similar function *k*(*x*) = *a* + *bx*^1.9^, which is close to the square of *x* rather than the cubic of *x*. This is probably because the shared region of the two solvent spheres after excluding the two atoms’ regions has torus- or disk-like shapes, as discussed in the Methods section.

Second, the proposed algorithm takes log-linear time to the number of atoms involved in a CSAV calculation as discussed in our time complexity analysis in the Methods section. Note that the least-square fits of the log-linear function in Figs 7, 8(A) and 8(B) are all determined independently and from different data setups. Note that all points are well aligned to their own log-linear functions. The three least-square fits have similar coefficients because all experiments are performed in the same workstation. This clearly shows that the proposed algorithm takes *O*(*n* log *n*) time, where *n* is the number of atoms involved in a CSAV calculation.

Last, the time complexity of the proposed sweep-line-based method is superior to that of the voxel-based method. In Fig 8(B), the running time *y* of the voxel-based method approximated using the least-square fit shows *y* ∝ *d*^4.62^, where *d* is the thickness of the solvent layer. It is similar to our expectation of its time complexity *O*(*d*^3^ *n*), where *n* is the number of atoms involved in the CSAV computation. In detail, the expected time complexity *O*(*d*^3^ *n*) approximates to *O*(*n*^4.88^). This is because the total number of iterations in the for-loops in lines 5–7 of Algorithm 1 is proportional to *d*^3^ and it has shown that *n* ∝ *d*^1.88^ from the least square fit of *k* = 10.03 + 4.93*x*^1.88^. This clearly indicates that the proposed sweep-line-based method has the time complexity of *O*(*n* log *n*) and thus is more efficient than that of the voxel-based method.

#### Accuracy of and computational cost increase by resolution changes

**Fig 9:**
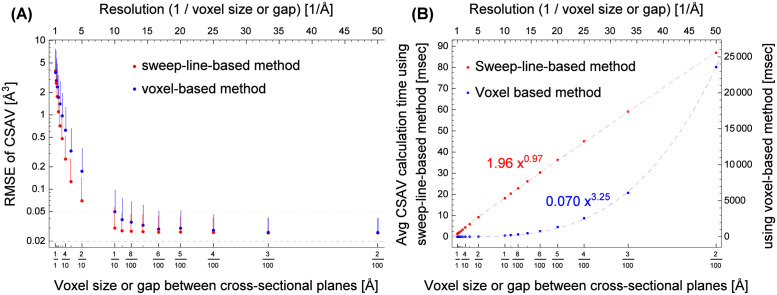
The comparison of (A) the root mean square error and (B) the computational cost in different resolutions determined using the sweep-line-based method (red) and the voxel-based method (blue).

**Fig 10:**
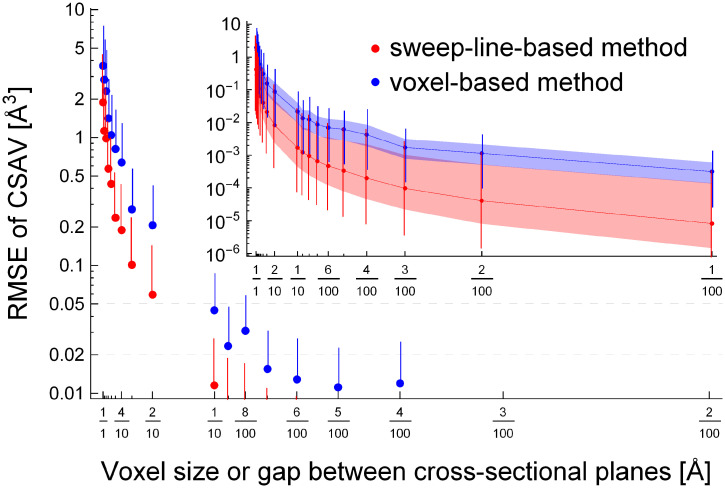
The comparison of the error distributions determined using the sweep-line-based method (red) and the voxel-based method (blue) by determining CSAVs of random systems in various gaps between cross-sectional planes or the voxel sizes.

Fig 9 compares the accuracy (or error) and the computational cost of the sweep-line-based method with those of the voxel-based method when the resolution increases (or the gap between cross-sectional planes and the size of voxels decrease). The results are determined from 3UC7 with a 3.5 Å thickness of the solvent layer and 18 different values ranging 0.02–1.0 Å for the gaps between cross-sectional planes or the voxel sizes: 0.02, 0.03, 0.04, 0.05, 0.06, 0.07, 0.08, 0.09, 0.1, 0.2, 0.3, 0.4, 0.5, 0.6, 0.7, 0.8, 0.9, and 1 Å. Fig 9(A) shows the decrease in error (or the increase in accuracy) of the CSAV as the resolution increases (or the gap between cross-sectional planes and the voxel size decrease). The error was measured using the root mean square error (RMSE) between the best approximation of a CSAV and the CSAV value determined using the sweep-line-based method (red) and the voxel-based method (blue). Since the true CSAV of two atoms cannot be determined because of the complex geometries of CSAVs, we estimate the true volume as follows. First, given two atoms, the lower (and upper) bound of their CSAV is determined by counting 0.02 × 0.02 × 0.02 Å^3^ voxels that are fully (and at least partially) enclosed in the CSAV, using the modified version of Algorithm 1. Then, we consider the average value of the lower and the upper bounds as the estimation of the true CSAV *v* of the two atoms. The error *e* of the CSAV $\hat{v}$ determined using the sweep-line-based method (red) or the voxel-based method (blue) is defined as the difference with the true CSAV *v*: $e=v-\hat{v}$. In the figure, each red point represents the root mean square error (RMSE) of the CSAV in the *y*-axis determined using the sweep-line-based method when the gap between cross-sectional planes is *x* Å; the red vertical bar connected to the point represents the range between RMSE and the 95th percentile of the absolute error |*e*|. In a similar manner, each blue point and its corresponding blue vertical bar are determined using the voxel-based method. Two gray dashed horizontal lines show the range of error values where the CSAV $\hat{v}$ determined using the sweep-line-based or voxel-based methods is converged enough to the estimation of the true CSAV *v*. The figure shows that the CSAVs determined using the sweep-line-based method are more accurate than those using the voxel-based method. However, it is difficult to compare the accuracy in the figure when the gap between cross-sectional planes or the voxel size in the *x*-axis is smaller than 1/10 Å (or the resolution is larger than 10 Å^–1^). This is primarily because the true CSAV value is approximated using the 0.02 × 0.02 × 0.02 Å^3^ voxels. The true accuracy between the two methods will be discussed again with Fig 10.

Fig 9(B) shows the increase in computation time as the resolution increases (or the gap between cross-sectional planes or the voxel size decreases). The figure is determined in a similar manner to Fig 9(A) except that the *y*-value of each point represents the averaged CSAV computation time. The pink (and light blue) dashed curve shows the least-square fit of *ax*^*b*^ to red (and blue) points. Each dashed curve shows the growing rate of the CSAV computation time as a function of the resolution *m*; the resolution is the inverse of the gap *δ* between cross-sectional planes or the inverse of the voxel size *δ*: *m* = 1/*δ*. The figure shows that the time complexity of the sweep-line-based method is *O*(*m*) and that of the voxel-based method is *O*(*m*^3^). This agrees with our time complexity analysis in the Methods section.

Fig 10 shows the decrease in *true* error (or the increase in accuracy) of the CSAV determined using the sweep-line-based and voxel-based methods as the resolution increases. Unlike Fig 9(A) where the true CSAVs are approximated using the smallest practical voxels, Fig 10 is determined using a closed-form solution that calculates the *true* CSAVs of simple random systems. To evaluate the accuracy of the two methods, we first generate a system that is composed of two random atoms with 3.5 Å thick solvent layers: each atom is at a random location, and its radius is randomly selected from the set {1.2 Å, 1.7 Å, 1.55 Å, 1.52 Å, 1.8 Å} whose element represents hydrogen, carbon, nitrogen, oxygen, or sulfur. The true CSAV value *v* of the random system is determined using the inclusion-exclusion principle and the closed-form solution calculating the intersection volume of two spheres. The random system is regenerated if *v* is smaller than 5 Å^3^. Given the random system, we determine the CSAV values $\hat{v}$ using the sweep-line-based method with a gap *δ* between cross-sectional planes (and using the voxel-based method with a voxel size of *δ* × *δ* × *δ* Å^3^ or simply *δ*). We use 19 different *δ* values ranging 0.01–1.0 Å for the gaps (and the voxel sizes). This process is repeated 30,000 times. From those, the statistics of error $e=v-\hat{v}$ are determined, including the root mean square error (RMSE) and the median, 5th, 25th, 75th, and 95th percentiles of the absolute error |*e*|. Fig 10 shows the error distributions determined from the random systems using the sweep-line-based method (red) and the voxel-based method (blue). Similar to Fig 9(A), the red and blue points and their corresponding vertical bars represent the RMSEs and the 95th percentiles of the absolute errors |*e*| determined using the sweep-line-based method with the gap of *x* between cross-sectional planes and the voxel-based method with the voxel size of *x*, respectively. The figure shows the RMSE range of [10^−2^, 10] only in the *y*-axis to make the comparison with Fig 9(A) easy. The inset shows the same results in a different view: the red (and blue) curve, the light red (and light blue) shaded region, and the red (and blue) vertical bars represent the median, the range of the 25th–75th percentiles, and the range of the 5th–95th percentiles of |*e*| determined using the sweep-line-based method (and the voxel-based method), respectively.

The plot shows that the sweep-line-based method is more accurate than the voxel-based method in all ranges of the gap or the voxel size *δ* in the *x*-axis. Recall that Fig 9(A) cannot clearly distinguish the accuracies of the two methods when *δ* < 1/10. This is primarily because the true CSAV value in Fig 9(A) is approximated using the 0.02 × 0.02 × 0.02 Å^3^ voxels. In Fig 10, the true CSAV is not approximated but directly calculated using the inclusion-exclusion principle and the closed-form solution. This enables to clearly compare the accuracies of the two methods even when *δ* is small. Note that the accuracies or error distributions of the two methods in Fig 9(A) are similar to those in Fig 10 when *δ* ∈ [1, 2/10]. This indirectly shows that if the true CSAVs in Fig 9(A) can be determined, the figure will show that the sweep-line-based method is clearly more accurate than the voxel-based method, like the inset in Fig 10. Our detailed inspection of Fig 10 shows that the sweep-line-based method is around 100-times more accurate than the voxel-based method in terms of the median of the absolute error.

Figs 9 and 10 shows that the proposed sweep-line-based method is superior to the voxel-based method in terms of both accuracy and computational efficiency. The resolution or the gap between cross-sectional planes controls the trade-off between the computational cost and the accuracy. The economical value of the gap between cross-sectional planes is 0.1 Å in our close inspection.

## Conclusion and discussion

In this work, we propose the sweep-line-based method that efficiently determines the common solvent accessible volume (CSAV). We define CSAV as the shared volume of two atoms’ solvent accessible (or interacting) regions excluding the volume occupied by atoms. The proposed method determines the CSAV by numerically integrating its cross-sectional area. In order to efficiently determine the area, the method divides the area into a list of disjoint solvent patches utilizing the sweep-line algorithm [29] and calculates the area of solvent patches using the proposed closed-form solution. Our results show that the proposed method finds the CSAV of two atoms in log-linear time *O*(*n* log *n*), where *n* is the number of atoms involved in the CSAV calculation. The resolution *m* is the model parameter that is the inverse of the gap between cross-sectional planes, which controls the trade-off between accuracy and computational cost. Our results show that the resolution of 10 Å^–1^ (or the gap of 0.1 Å) is an economical choice which balances good accuracy with low computational cost. Considering both *n* and *m*, the proposed sweep-line-based method finds the CSAV in *O*(*mn* log *n*) time. Our experiment shows that the number *n* of atoms involved in the CSAV calculation is near proportional to the square of the solvent layer thickness *d*: *n* ∝ *d*^2^. This is probably because the shared region of the two solvent spheres has torus- or disk-like shapes after excluding their corresponding atoms’ regions. The accuracy and the computational efficiency of the proposed sweep-line-based method are compared to those of the naïve voxel-based method. Our results show that the sweep-line-based method outperforms the voxel-based method in both accuracy and computational efficiency.

The proposed method can be a useful tool to measure the solvents’ mediation of protein-protein interactions. Proteins interact with other proteins mediated by solvents before they have direct contract, and often the hydrogen-bond networks in solvents play an important role in mediating the interactions between proteins [8, 9] At the atomic level, these solvent-mediated protein-protein interactions can be described as the springs (or spring tensors) added between two proteins’ atoms by solvents or the spring networks in solvents located between the two atoms. More specifically, the potential energy surface of solvated proteins can be approximated using the second-order Taylor expansion. Assume that solvent particles *s* influence the interaction between two atoms *a* and *b* of proteins. The spring (or spring tensor) between atoms *a* and *b* influenced by solvent particles *s* can be determined from the second-order term of the Taylor expansion, as follows: [32]

$$\begin{array}{c} \delta_{a}^{⊤}H_{ab}^{\prime }\delta_{b}=\delta_{a}^{⊤}\left(H_{ab}-H_{as}H_{ss}^{-1}H_{sb}\right)\delta_{b}\;, \end{array}$$ (4)

where ***δ***_*a*_ and ***δ***_*b*_ are the location changes of *a* and *b*, respectively, **H**_*ab*_, **H**_*as*_, and **H**_*sb*_ indicate the springs (or spring tensors) between *a* and *b*, between *a* and *s*, and between *s* and *b*, respectively, and $H_{ss}^{-1}$ determines the dynamics of the solvent particles *s*. In the above, if the second summand $H_{as}H_{ss}^{-1}H_{sb}$ can be determined correctly with considering all possible and realistic solvent particle locations, we can consider the summand as an ideal implicit solvent model, which is infeasible to determine. Here, the proposed method determining the CSAV can help predict the summand. Consider *a* and *b* effectively interact with solvent particles in their own solvent spheres. We can approximate the ideal implicit solvent model $H_{as}H_{ss}^{-1}H_{sb}$ using the following integration:

$$\begin{array}{c} \int_{V(a,b)}H_{au}f\left(u\right)H_{ub}\;du\;, \end{array}$$ (5)

where *f*(*u*) predicts the dynamics of a solvent particle at location *u*, and V(a,b) is the region of the CSAV of two atoms *a* and *b*. The above integration can be solved utilizing the proposed method by replacing Eqs (1)–(3): the CSAV considers **H**_*au*_ *f*(*u*)**H**_*ub*_ = 1. In other words, the proposed method determining the CSAV can be utilized to measure how much the solvent at *u* can mediate the spring interaction between *a* and *b* through its dynamics *f*(*u*).

There are many methods designed to measure the solvent accessible surface/volume of a protein [11–26], which are often used in implicit solvent models to describe the forces exerted on atoms on the protein surface by the solvent [1–7]. However, the existing methods have limitations in solving the above integration problem in Eq (5). First, it is impractical to use the existing methods to determine the interval V(a,b), representing the region of the CSAV. Determining the solvent accessible volume or the protein volume of an entire protein can be solved by finding the union of spheres representing solvent spheres and atoms, while determining the CSAV requires both intersection and exclusion of spheres representing solvent spheres and atoms. Determining the CSAV is theoretically possible using the inclusion-exclusion principle and the existing methods solving the union, but this may require an impractically large amount of computations in the worst-case scenarios. Second, the existing methods cannot apply the indefinite integral ∫ **H**_*au*_ *f*(*u*)**H**_*ub*_ *du* without appropriate modifications on the methods.

The proteins are surrounded by two groups of solvents: the hydration shell and bulk solvent. The hydration shell is about 3.5 Å thick layer of solvent molecules next to the surface of proteins [10]. The low-frequency global motions of proteins assist their conformational changes, and therefore they are related to the functions of the proteins. Our previous study shows that the protein structures, the hydration shell, and bulk solvent has about 50%, 35%, and 15% contributions to the low-frequency motions, respectively [32]. This implies that we may need to use a solvent layer whose thickness is greater than 3.5 Å, such as 8 Å, to include the contributions by the hydration shells and the bulk solvents and thus to achieve better accuracy when evaluating the interaction between proteins bridged or mediated by solvents. The proposed sweep-line-based method can be extended to efficiently measure the volumes of solvent layers with different thicknesses by simply adding multiple solvent spheres, which is the additional advantage of the proposed sweep-line-based method.

## Supporting information

S1 File The program source code implementing the sweep-line-based method that determines the common solvent accessible volume of two atoms. (ZIP)

## References

[1] Qiu D, Shenkin PS, Hollinger FP, Still WC. The GB/SA Continuum Model for Solvation. A Fast Analytical Method for the Calculation of Approximate Born Radii. J Phys Chem A. 1997;101(16):3005–3014. doi: 10.1021/jp961992r

[2] Fogolari F, Brigo A, Molinari H. Protocol for MM/PBSA Molecular Dynamics Simulations of Proteins. Biophys J. 2003;85:159–166. doi: 10.1016/S0006-3495(03)74462-2 12829472 PMC1303073

[3] Still WC, Tempczyk A, Hawley RC, Hendrickson T. Semianalytical treatment of solvation for molecular mechanics and dynamics. J Am Chem Soc. 1990;16:6127–6129. doi: 10.1021/ja00172a038

[4] Kleinjung J, Fraternali F. Design and application of implicit solvent models in biomolecular simulations. Curr Opin Struct Biol. 2014;25:126–134. doi: 10.1016/j.sbi.2014.04.003 24841242 PMC4045398

[5] Wagoner JA, Baker NA. Assessing implicit models for nonpolar mean solvation forces: The importance of dispersion and volume terms. Proc Natl Acad Sci USA. 2006;103(22):8331–8336. doi: 10.1073/pnas.0600118103 16709675 PMC1482494

[6] Mongan J, Simmerling C, McCammon JA, Case DA, Onufriev A. Generalized Born Model with a Simple, Robust Molecular Volume Correction. J Chem Theory Comput. 2007;3(1):156–169. doi: 10.1021/ct600085e 21072141 PMC2975579

[7] Lazaridis T, Karplus M. Effective energy function for proteins in solution. Proteins. 1999;35:133–52. doi: 10.1002/(SICI)1097-0134(19990501)35:2<133::AID-PROT1>3.0.CO;2-N 10223287

[8] Ahmad M, Gu W, Geyer T, Helms V. Adhesive water networks facilitate binding of protein interfaces. Nat Commun. 2011;2(261):1–7. doi: 10.1038/ncomms1258 21448160

[9] Koga K, Zeng XC, Tanaka H. Solvent-induced interactions between hydrophobic and hydrophilic polyatomic sheets in water and hypothetical nonpolar water. J Chem Phys. 1997;106:9781–9792. doi: 10.1063/1.473867

[10] Fogarty AC, Duboué-Dijon E, Sterpone F, Hynesac JT, Laage D. Biomolecular hydration dynamics: a jump model perspective. Chem Soc Rev. 2013;40:5672–5683. doi: 10.1039/c3cs60091b 23612685

[11] Richards FM. The interpretation of protein structures: total volume, group volume distributions and packing density. J Mol Biol. 1974;82(1):1–14. doi: 10.1016/0022-2836(74)90570-1 4818482

[12] Gerstein M, Tsai J, Levitt M. The volume of atoms on the protein surface: calculated from simulation, using Voronoi polyhedra. JMB. 1995;249:955–966. doi: 10.1006/jmbi.1995.0351 7540695

[13] Richards FM. Areas, volumes, packing, and protein structure. Ann Rev Biophys Bioeng. 1977;6:151–176. doi: 10.1146/annurev.bb.06.060177.001055 326146

[14] Connolly ML. Computation of molecular volume. J Am Chem Soc. 1985;107:1118–1124. doi: 10.1021/ja00291a006

[15] Petitjean M. On the Analytical Calculation of van der Waals Surfaces and Volumes: Some Numerical Aspects. J Comput Chem. 1994;15(5):507–523. doi: 10.1002/jcc.540150504

[16] Pavanï R, Ranghino G. A method to compute the volume of molecule. Computers and Chemistry. 1982;6:133–135. doi: 10.1016/0097-8485(82)80006-5

[17] Gibson KD, Scheraga HA. Volume of the intersection of three spheres of unequal size: a simplified formula. J Phys Chem. 1987;91:4121–4122. doi: 10.1021/j100299a035

[18] Rowlinson JS. The triplet distribution function in a fluid of hard spheres. Molecular Physics. 1963;6:517–524. doi: 10.1080/00268976300100581

[19] Kang YK, Nemethy G, Scheraga HA. Free energies of hydration of solute molecules. 1. Improvement of the hydration shell model by exact computations of overlapping volumes. J Phys Chem. 1987;91(15):4105–4109. doi: 10.1021/j100299a032

[20] Richmond TJ. Solvent accessible surface area and excluded volume in proteins: Analytical equations for overlapping spheres and implications for the hydrophobic effect. J Mol Biol. 1984;178:63–89. doi: 10.1016/0022-2836(84)90231-6 6548264

[21] Hayryan S, Hu CK, Skřivánek J, Hayryane E, Pokorný I. A New Analytical Method for Computing Solvent-Accessible Surface Area of Macromolecules and its Gradients. J Comput Chem. 2005;26:334–343. doi: 10.1002/jcc.20125 15643653

[22] Rashin AA, Iofin M, Honig B. Internal cavities and buried waters in globular proteins. Biochemistry. 1986;25:3619–3625. doi: 10.1021/bi00360a021 3718947

[23] Müller JJ. Calculation of scattering curves for macromolecules in solution and comparison with results of methods using effective atomic scattering factors. J Appl Cryst. 1983;16:74–82. doi: 10.1107/S0021889883009978

[24] Pavlov MY, Fedorov BA. Improved technique for calculating X-ray scattering intensity of biopolymers in solution: Evaluation of the form, volume, and surface of a particle. Biopolymers. 1983;22:1507–1522. doi: 10.1002/bip.360220607

[25] Chen CR, Makhatadze GI. ProteinVolume: calculating molecular van der Waals and void volumes in proteins. BMC Bioinformatics. 2015;16. doi: 10.1186/s12859-015-0531-2 PMC4379742 25885484

[26] Georgiev GD, Dodd KF, Chen BY. Precise parallel volumetric comparison of molecular surfaces and electrostatic isopotentials. Algorithms Mol Biol. 2020;15(11). doi: 10.1186/s13015-020-00168-z 32489400 PMC7247173

[27] Kang YK, Nemethy G, Scheraga HA. Free energies of hydration of solute molecules. 2. Application of the hydration shell model to nonionic organic molecule. J Phys Chem. 1987;91(15):4109–4117. doi: 10.1021/j100299a032

[28] Kang YK, Nemethy G, Scheraga HA. Free energies of hydration of solute molecules. 3. Application of the hydration shell model to charged organic molecules. J Phys Chem. 1987;91(15):4118–4120. doi: 10.1021/j100299a032

[29] Shamos MI, Hoey D. Geometric intersection problems. In: 17th Annual Symposium on Foundations of Computer Science (SFCS 1976). Houston, TX, USA, USA: IEEE; 1976. p. 208–215.

[30] Ponder JW, Richards FM. An efficient newton-like method for molecular mechanics energy minimization of large molecules. J Comput Chem. 1987;8:1016–1024. doi: 10.1002/jcc.540080710

[31] MacKerell AD, Bashford D, Bellott, Dunbrack RL, Evanseck JD, Field MJ, et al. All-Atom Empirical Potential for Molecular Modeling and Dynamics Studies of Proteins. J Phys Chem B. 1998;102(18):3586–3616. doi: 10.1021/jp973084f 24889800

[32] Majumdar AB, Kim I, Na H. Effect of solvent on protein structure and dynamics. Phys Biol. 2020;17(3):036006. doi: 10.1088/1478-3975/ab74b3 32040946

