# Supplementary material for: An efficient algorithm calculating common solvent accessible volume
Source: PLoS One. 2022 Mar 21;17(3):e0265614. doi: 10.1371/journal.pone.0265614 (PMC8936456; doi:10.1371/journal.pone.0265614)
Supplement: Attachment — Submitted filename: PONE-D-21-21492-review-reply.pdf [file pone.0265614.s002.pdf]

Dear Dr. Na,

Thank you for submitting your manuscript to PLOS ONE. After careful consideration, we feel that it has merit but does not fully meet PLOS ONE's publication criteria as it currently stands. Therefore, we invite you to submit a revised version of the manuscript that addresses the points raised during the review process.

Response: The authors thank the editor and reviewers for the valuable recommendations, suggestions, and comments.

Please make sure that you compare your method with the current state of the art similar methods.

Response: There are no existing methods that can be used to determine the common solvent accessible volume (CSAV). We explained this in the Introduction section and the Conclusion and Discussion section on pages 3 and 19-20, respectively.

The existing methods determining the solvent accessible volume (SAV) of an entire protein cannot be used to determine the CSAV. This is because the SAV calculation can be solved (or considered as) using only the union operation of spheres (conceptually, union volume of all solvent-interacting spheres - union volume of all atoms), while the CSAV calculation requires both intersection and exclusion operations of spheres. The output of intersection and exclusion operations can be determined theoretically using the inclusion-exclusion principle and union operations. However, this approach is *impractical* because the inclusion-exclusion principle with n sets (or spheres) has $2^n - 1$ number of terms. In other words, calculating the CSAV using the existing methods (determining union of spheres) will require the union of all different combinations of spheres (2^n number of unions) in the worst-case scenarios.

This is why we compare the proposed sweep-line-based method with a naïve voxel-based method that is designed to determine the CSAV.

And please make sure that you use an independent dataset to assess the performance.

Response: Thank you very much for your recommendation. We replaced our dataset. Now, we use 52 proteins, where 50 of them are obtained from the list of proteins used in another paper (G. D. Georgiev *et al*. published to Algorithms Mol. Biol. in 2020). The new dataset still contains the 2 proteins used in our previous dataset: Ubiquitin (1UBQ) and Trp-cage (3UC7, one of the smallest protein structures). This is described on page 12.

Please submit your revised manuscript by Jan 09 2022 11:59PM. If you will need more time than this to complete your revisions, please reply to this message or contact the journal office at plosone@plos.org. When you're ready to submit your revision, log on to https://www.editorialmanager.com/pone/ and select the 'Submissions Needing Revision' folder to locate your manuscript file.

[Note: HTML markup is below. Please do not edit.]

Reviewers' comments:

Reviewer's Responses to Questions

Comments to the Author

1. Is the manuscript technically sound, and do the data support the conclusions?

The manuscript must describe a technically sound piece of scientific research with data that supports the conclusions. Experiments must have been conducted rigorously, with appropriate controls, replication, and sample sizes. The conclusions must be drawn appropriately based on the data presented.

Reviewer #1: Partly

Reviewer #2: Partly

---

2. Has the statistical analysis been performed appropriately and rigorously?

Reviewer #1: No

Reviewer #2: Yes

---

3. Have the authors made all data underlying the findings in their manuscript fully available?

The PLOS Data policy requires authors to make all data underlying the findings described in their manuscript fully available without restriction, with rare exception (please refer to the Data Availability Statement in the manuscript PDF file). The data should be provided as part of the manuscript or its supporting information, or deposited to a public repository. For example, in addition to summary statistics, the data points behind means, medians and variance measures should be available. If there are restrictions on publicly sharing data—e.g. participant privacy or use of data from a third party—those must be specified.

Reviewer #1: Yes

Reviewer #2: No

---

4. Is the manuscript presented in an intelligible fashion and written in standard English?

PLOS ONE does not copyedit accepted manuscripts, so the language in submitted articles must be clear, correct, and unambiguous. Any typographical or grammatical errors should be corrected at revision, so please note any specific errors here.

Reviewer #1: Yes

Reviewer #2: Yes

---

5. Review Comments to the Author

Please use the space provided to explain your answers to the questions above. You may also include additional comments for the author, including concerns about dual publication, research ethics, or publication ethics. (Please upload your review as an attachment if it exceeds 20,000 characters)

Reviewer #1: The algorithm is interesting. However, the motivation that is given is to apply it to proteins, but I dont see any attempt to compare the results of CSAV to exiting methods. Are the values different numerically for a set 21 proteins that was used to benchmark the speed etc.?

Response: Yes. Each CSAV value and its calculation time are different. The CSAV value is determined by (or depends on) its geometry: two solvent spheres, involved-atoms, and their locations and sizes. The computation time depends on the geometry and additionally the computing environments, such as CPU speed and tasks running on the computer. In our results, the primary purpose of showing the running time is i) to show the agreement between our running time analysis and the actual running time of the proposed method and ii) to compare the computational efficiency of the sweep-line-based method with that of the voxel-based method.

We re-ran the entire dataset by setting the priority of each program high. As a result, the coefficients of running time decreased. We additionally show three digits of the coefficients in our updated plots. Now, the coefficients are updated from 0.07 to 0.063, 0.064 and 0.066 in Fig 7, Fig 8(a) and Fig 8(b), respectively. We did not re-run our program to collect data points for Fig 9 since the computation of the voxel-based method takes too much time.

Faster is not better for protein science. Statement is made that CSAV is more accurate. How can one argue that the algorithm is more accurate without providing the criteria for it?

Response: As we responded to the editor's recommendation, there are no practical and existing methods that can be used to determine CSAV and thus can be compared with our proposed sweep-line-based method. Therefore, to state that the proposed method is accurate, we compared the accuracy of the proposed sweep-line-based method with that of the naïve voxel-based method.

To provide a more solid argument, we added a new experiment (Fig. 10) that compares the *true* errors determined by the two methods. The true errors are evaluated with the *true* CSAV values that are calculated using a closed-form solution of the simple random systems; if the geometry of a system is simple enough, its CSAV can be algebraically calculated. Our new results show that the sweep-line-based method is clearly (around 100 times) more accurate than the voxel-based method. We added the description regarding Fig 10 on pages 17-18. Please refer to the table below in this reply to reviewer 2 for the additional details.

So if any statement about importance for biology is stripped from the manuscript, it is a computer science paper calculating the volumes of collection of spheres of arbitrary shapes. From that perspective it is probably OK but i still think it must be critically compared to other methods. If, however, any mention of biology remains in the manuscript, real comparison with the existing methods must be done. After that is done, a compelling evidence (other than speed of calculations and scaling with the size) must be made why CSAV is better in providing physical insights into protein structure analysis.

Response: As we responded to the editor's recommendation, there are no practical and existing methods that can be used to determine the CSAV. We explained this on pages 3 and 19-20.

Regarding the compelling evidence, we added a discussion explaining how the proposed method can provide a physical insight into protein study. The proposed sweep-line-based method can be used to predict the amount of the spring interaction between two atoms of proteins that is influenced by solvents near the two atoms (or within their solvent-accessible spheres). This prediction can be made by replacing the equations (1)-(3) with a different function that integrates the values of points in the CSAV. We explained the detailed discussion in the second paragraph (including equations (4) and (5)) of the Conclusion and Discussion section on page 19.

Reviewer #2: In this work, Kim and Na describes a new method to calculate the solvent accessibility of a protein-protein interface. Their method differentiates itself from the others in the way that it takes both protein parties into account during the calculation.

Response: We apologize that we did not clarify how the experiments were performed. Actually, the proposed method can determine the CSAV between atoms in different proteins. However, our work focuses on evaluating the accuracy and the efficiency of the proposed method, and thus we performed the evaluation by determining the CSAVs of atom pairs in *one* protein. We clarified this on page 13.

Although this idea is novel and worth exploring, the way the authors presented their research requires a serious reconsideration. The main critical points are listed below.

- The authors indicated that:

"The source code of the proposed sweep-line-based method will be available from GitHub repository when this paper is published: https://github.com/htna/CSAV."

I do not understand, why the Github repo is not presented together with this version of the paper. The code and some example cases should be presented with the paper at the submission stage.

Response: Thank you for understanding the value of our study.

Regarding uploading our source code into Github, we planned to share the source code after our paper is published since we were concerned that (maybe) others can publish the same work to ours after regenerating the results from our source code (as if it is their original work). We now provide our source code as Supporting Information, and so you can look at the source code. We plan to upload our source code into Github after the paper is accepted; the dataset (protein structures) and the list of atom pairs and their corresponding CSAV value calculated using the proposed sweep-line-based method are on Github already.

- The authors tested their methods on 21 crystal structures. Why are these particular structures selected? What are the functions of these structures, size of their monomers, shapes, secondary structure content, stoichiometry, etc. What is the biological relevance of using these cases? Are there already any water molecules around their interfaces?

Response: The 21 structures in our previous dataset were selected only by considering their size variances rather than their functional importance. Additionally, we tried to include alpha, beta, and alpha+beta proteins in the dataset. This is because the CSAV value between two atoms is determined and calculated only from their geometry (the sizes and locations of the solvent-accessible spheres and the atoms to be excluded from the CSAV). As responded to the editor’s recommendation, we replaced our previous dataset containing the 21 structures with a new dataset containing 52 proteins obtained from the list of proteins used by G. D. Georgiev *et al*. (published to Algorithms Mol. Biol. in 2020) This is described in the Dataset preparation section on page 12.

Regarding the existence of water molecules, we used only protein atoms while discarding other molecules, such as waters and ligands, in our CSAV calculation. We clarified this on page 12.

Also, why didn't the author consider using a larger set? Most importantly, what are CSAV values of the probed data set?

Response: We now use a larger dataset containing 52 proteins, as mentioned above. The total number of CSAVs (atom pairs) determined from the 52 proteins is 2,267,179. This is described on page 13. We uploaded our new dataset and the CSAV values of atom pairs into GitHub, which are determined with a 3.5 Å thickness of the solvent layer and a 0.1 Å gap between cross-sectional planes.

How are these values compared to the standard algorithms used in the field? How fast is the algorithm compared to to these standard algorithms, etc.?

Response: As we responded to the editor's recommendation, there are no standard methods determining the CSAV. Therefore, we compared the efficiency and accuracy of the proposed method with those of the voxel-based method.

It is difficult to provide a specific number that shows the computational speedups of the sweep-plane-based method against the voxel-based method since their running times depend on the gap between cross-sectional planes and the voxel size. However, in my opinion, the below table will give you an idea of how much the proposed method is more efficient and accurate compared to the voxel-based method.

The following table is based on Fig 9(B) determined from 3UC7 (Trp-Cage) and Fig 10 (simple random systems). In the table, the first column (delta) shows the gap between cross-sectional planes or the size of voxels, and the second and third columns compare the average running times of determining CSAV values in milliseconds, the fourth and fifth columns compare the accuracies in terms of the median value of absolute error, and the sixth column shows the accuracy ratio between the two methods. The sixth column seems to show that the sweep-plane-based method is about 100 times more accurate than the voxel-based method when the gap or voxel size is small enough. We were not able to compute the running time of the voxel-based method with 0.01 Å voxel size (the last row of the third column) since this takes too much time to finish its computation (several weeks).

delta	running time in Fig 9(B) [msec]		median of absolute error in Fig 10 [Å^3]		
	sweep-plane	voxel	sweep-plane (x)	voxel (y)	(y)/(x)
1.0	1.38027	0.00156006	0.175055	3.78768	21.6371
0.9	1.63612	0.00429017	0.0787716	2.60861	33.1161
0.8	1.91751	0.0181357	0.0462054	1.61197	34.887
0.7	2.25293	0.0694228	0.0195679	0.675141	34.5025
0.6	2.71041	0.304212	0.0114967	0.362252	31.5092
0.5	3.38787	0.945593	0.00396393	0.191313	48.2635
0.4	4.38904	2.30246	0.0016073	0.0946459	58.885
0.3	5.93604	6.06611	0.000440644	0.0242149	54.9534
0.2	9.25059	21.2233	0.0000691563	0.00775076	112.076
0.1	18.2264	166.879	2.80942*10^-6	4.71918*10^-4	167.977
0.09	20.3559	229.697	1.48823*10^-6	1.82103*10^-4	122.362
0.08	22.8643	324.278	8.87662*10^-7	1.53215*10^-4	172.605
0.07	26.1642	485.876	4.25374*10^-7	7.62154*10^-5	179.173
0.06	30.3405	768.919	2.17787*10^-7	4.83549*10^-5	222.028
0.05	36.2494	1335.91	1.13778*10^-7	3.88671*10^-5	341.605
0.04	45.0649	2568.73	3.97514*10^-8	1.82186*10^-5	458.313
0.03	59.1229	6097.54	9.32011*10^-9	2.90304*10^-6	311.481
0.02	86.922	23577.1	1.64197*10^-9	1.28894*10^-6	784.996
0.01			6.70718*10^-11	9.86444*10^-8	1470.73

- The authors talk about accuracy, RMSE, etc., however as they cannot benchmark their results against an experimental data set, I do not think that the use of these terms is proper.

Response: The RMSE (root mean square error) is a widely used term to compare the accuracy of any values. Since it is very challenging (or taking too much time to get the true CSAV value when the protein size is large), we evaluated RMSE in Fig 9(A) with the best approximation of CSAV that is determined using the $0.02 \times 0.02 \times 0.02$ Å voxels. To overcome this limitation of the accuracy comparison, we added Fig 10 that compares the accuracies of the two methods with the true CSAV values of simple systems (having a closed-form solution). The description of Fig 10 is on pages 17-18.

All in all, the paper should discuss not only the architecture and implementation of the algorithm, but also the algorithm's usefulness from the biology perspective, as well as its efficiency compared to the existing methods.

Response: We discuss the usefulness of our algorithm in computational biology in the Conclusion and Discussion section on page 19.

As we responded to the editor's recommendation, there are no existing methods that can be used to determine CSAV. We explained this on pages 3 and 19-20.

---

6. PLOS authors have the option to publish the peer review history of their article (what does this mean?). If published, this will include your full peer review and any attached files.

If you choose “no”, your identity will remain anonymous but your review may still be made public.

Do you want your identity to be public for this peer review? For information about this choice, including consent withdrawal, please see our Privacy Policy.

Reviewer #1: No

Reviewer #2: No

[NOTE: If reviewer comments were submitted as an attachment file, they will be attached to this email and accessible via the submission site. Please log into your account, locate the manuscript record, and check for the action link "View Attachments". If this link does not appear, there are no attachment files.]
